# The association between essential trace element (copper, zinc, selenium, and cobalt) status and the risk of early embryonic arrest among women undergoing assisted reproductive techniques

**DOI:** 10.3389/fendo.2022.906849

**Published:** 2022-10-26

**Authors:** Yu Cao, Chunmei Liang, Lingchao Shen, Zhikang Zhang, Tingting Jiang, Danyang Li, Weiwei Zou, Jieyu Wang, Kai Zong, Dan Liang, Dongmei Ji, Yunxia Cao

**Affiliations:** ^1^ Department of Obstetrics and Gynecology, The First Affiliated Hospital of Anhui Medical University, Hefei, Anhui, China; ^2^ National Health Commission (NHC) Key Laboratory of Study on Abnormal Gametes and Reproductive Tract, Anhui Medical University, Hefei, Anhui, China; ^3^ Key Laboratory of Population Health Across Life Cycle (Anhui Medical University), Ministry of Education of the People’s Republic of China, Hefei, Anhui, China; ^4^ Anhui Province Key Laboratory of Reproductive Health and Genetics, Hefei, Anhui, China; ^5^ Anhui Provincial Engineering Research Center of Biopreservation and Artificial Organs, Hefei, Anhui, China; ^6^ Anhui Provincial Institute of Translational Medicine, Hefei, Anhui, China; ^7^ School of Public Health, Anhui Medical University, Hefei, Anhui, China; ^8^ Technical Center of Hefei Customs District, Hefei, Anhui, China

**Keywords:** combined effects, early embryonic arrest, BKMR models, essential trace element, mixtures

## Abstract

**Background:**

Early embryonic arrest (EEA) leads to repeated cessation of fresh cycles among infertile women undergoing *in vitro* fertilization (IVF). Whether the levels of some essential trace elements [copper (Cu), zinc (Zn), selenium (Se) and cobalt (Co)] in the bodies of women are related to the risk of EEA warrants study.

**Objective:**

Our study aimed to investigate the associations of peripheral blood levels of Cu, Zn, Se, and Co and their mixtures with the risk of EEA.

**Methods:**

A total of 74 EEA cases (123 IVF cycles) and 157 controls (180 IVF cycles) from the reproductive center of the First Affiliated Hospital of Anhui Medical University in Hefei, China, between June 2017 and March 2020 were included in our study. Demographic and clinical data were collected from electronic medical records. Cu, Zn, Se, and Co levels were measured in blood samples collected on the day of oocyte retrieval when infertile women entered clinical treatment for the first time using an inductively coupled plasma mass spectrometer (ICP−MS). Generalized estimating equation (GEE) models were used to evaluate the associations of four essential trace element concentrations individually with the risk of EEA, and Bayesian kernel machine regression (BKMR) was used to explore the associations between four essential trace element mixtures and the risk of EEA.

**Results:**

Se concentrations of infertile women were significantly lower in the case group compared with the control group. Co levels were significantly higher in the case group compared with the control group. The differences in Cu and Zn concentrations between the two groups were not significant. Based on single-metal models, Co was positively associated with the risk of EEA before and after adjustment for all confounders (odd ratio (OR) = 1.72, 95% confidence interval (CI): 1.18−2.52; OR = 2.27, 95% CI: 1.37−3.77, respectively), and Se was negatively associated with the risk of EEA before adjustment for all confounders (OR = 0.18, 95% CI: 0.07−0.51). BKMR analyses showed that Se was significantly and negatively associated with the risk of EEA when all the other three metals (Cu, Zn, and Co) were fixed at the 25th, 50th, or 75th percentiles, whereas Zn displayed a significant and positive association with the risk of EEA when all the other three metals (Cu, Se and Co) were fixed at the 25th, 50th, or 75th percentiles. Co did not show any effect on the risk of EEA when all the other metals (Cu, Zn, and Se) were fixed at the 25th, 50th, or 75th percentiles. In addition, an increasing trend of the joint effect of four essential trace elements on the risk of EEA was found, although it was not statistically significant.

**Conclusion:**

The levels of essential trace elements (Cu, Zn, Se, and Co) might correlate with the risk of EEA to some extent. The present study might provide a real-world perspective on the relationship between essential trace elements and the risk of EEA when considering them as a single element or as mixtures.

## Introduction

Globally, 15% of couples suffer infertility worldwide, which is equivalent to 48.5 million couples. In addition, the prevalence change depends on the geographical region (1). In 2017, *in vitro* fertilization (IVF) accounted for 99% of all assisted reproductive technologies (ARTs) used in America (2); only 42% of cycles could achieve clinical pregnancies. It is within the best interest of the clinical community to maximize live birth rates from IVF, to prevent the attendant financial costs (3), psychological stress (4), and health concerns (5). Therefore, exploring risk factors affecting the success of ART is of great importance.

Early embryonic arrest (EEA), which is defined as the stagnation of the growth of early embryos cultured *in vitro* at the four- to eight-cell stage and the cessation of development, is one of the major causes of the recurrent failure of IVF cycles (6). Previous studies have found that four- to eight-cell embryos are in the phase of zygotic gene activation (7), a stage in which some mechanisms are being established and transformed. Some studies found that adverse environmental and genetic factors may lead to embryo stagnation at the four- to eight-cell stage and EEA occurrence, such as chromosomal aneuploidies (8), gene mutations or deletions (9), and maternal obesity (10).

To date, limited studies have been conducted on the relationship between the status of essential trace elements in the human body and IVF outcomes, even though appropriate levels of essential trace elements are seemingly needed for the success of ART (11). For instance, Wu et al. found that seminal Se levels were positively associated with pregnancy and live birth, and female serum Se levels were positively associated with blastocyst development (12). Another study in the USA identified unexpected negative associations between follicular fluid (FF) Zn and the proportion of oocytes fertilized, whereas higher FF Co levels were associated with a lower average embryo cell number per woman (13). Zinc (Zn), selenium (Se), copper (Cu), and cobalt (Co) accomplish decisive functions to maintain human health (12, 13) and play significant roles in human and mammalian reproduction. However, data describing the association of the status of essential trace elements with IVF outcomes, especially EEA, are very limited.

Therefore, in the present study, we aimed to investigate the associations of peripheral blood concentrations of four essential trace elements (Cu, Zn, Se, and Co) and their mixtures with the risk of EEA to provide a real-world perspective on the relationship between essential trace elements and EEA.

## Materials and methods

### Study design and participants

Couples with infertility were recruited from June 2017 to March 2020 at the Reproduction Centre of the First Affiliated Hospital of Anhui Medical University in Hefei, Anhui Province, China, to participate in this case−control study. The case group was defined based on the following criteria (1): aged between 20 and 40 years (2); EEA occurred in at least one IVF or ICSI treatment cycle; (3) both couples had no reported familial genetic factors and normal chromosomes; (4) the male partner’s sperm was of good quality; and (5) no donor oocytes or sperm were used in the ART process. The control group was matched for age and BMI with the same criteria as the case group, except for embryo quality, who had normal early embryo development and obtained at least 70% high-quality blastocysts in each oocyte retrieval cycle. Finally, 74 cases undergoing 123 cycles and 157 controls undergoing 180 cycles were included in this study. We obtained information about the demographic characteristics, clinical features, and medical history of the participants from the hospital electronic health system.

### IVF procedure and outcome assessment

The IVF process occurred as follows. First, women underwent one type of controlled ovarian hyperstimulation (COH) protocol, including long, short, gonadotropin-releasing hormone antagonist or microstimulation protocols, which were based on their ovarian response function and ages. Second, ovulation was triggered with human chorionic gonadotropin (hCG; 10,000 IU) when a minimum of two follicles had matured (diameter ≥18 mm). Third, after 36 h, oocyte retrieval was performed, and fresh semen was collected on the same day. Finally, the oocytes were fertilized by conventional insemination or ICSI based on clinical indications.

The numbers of retrieved mature oocytes (metaphase II, MII), oocytes with two pronuclei, and high-quality embryos was assessed after oocyte retrieval and fertilization. The Gardner system was used to evaluate blastocyst quality. Blastocysts of grade ≥4 BB on Days 5–6 were designated as high-quality blastocysts. To ensure high intra- and interrater reliabilities, we assigned two fixed embryologists to evaluate the quality of each embryo simultaneously.

### Sample collection and exposure assessment

Fasting anticoagulant blood samples from infertile women on the day of oocyte retrieval when they started clinical treatment for the first time were stored at −80°C until further assessment. Then, 500 μl HNO_3_ and a 10 mg/L gold (Au) solution (50 μl) were added to 100 μl of blood after the blood was thawed. The above mixture was digested for at least 1 h at 100°C. The completely digested mixture was diluted 1:50 with 0.05% Triton X-100.

The blood concentrations of Cu, Zn, Se, and Co were determined by ICPMS (Perkin Elmer NexION 350X, Shelton, CT, USA) simultaneously. In brief, ^65^Cu, ^66^Zn, ^78^Se, and ^59^Co were used as the isotopes; a multiple-element mixture standard stock solution (10 μg/ml, Perkin Elmer, USA, N9300233) and a multiple internal standard mixture solution (10 μg/ml) (Perkin Elmer, USA, N9303832) were used during determination. Multielement standard curves were prepared by serially diluting the standard stock solution. Finally, calibration curve details were as follows: 0.0 μg/L, 10.0 μg/L, 50.0 μg/L, 100.0 μg/L, and 200.0 μg/L for Cu; 0.0 μg/L, 50.0 μg/L, 100 μg/L, 200 μg/L, and 300 μg/L for Zn; 0.0 μg/L, 1.0 μg/L, 2.0 μg/L, 5.0 μg/L, 10.0 μg/L, and 20 μg/L for Se; and 0.0 μg/L, 1.0 μg/L, 2.0 μg/L, 5.0 μg/L, and 10.0 μg/L for Co. The kinetic energy discrimination mode was used for four elements. Sc was used as the internal standard element for the above analytes, and the final concentration of the internal standard was 20.0 μg/L.

The limits of detection (LODs) of Cu, Zn, Se, and Co were 0.136 μg/L, 0.845 μg/L, 0.575 μg/L, and 0.008 μg/L, respectively. The recovery rates of Cu, Zn, Se, and Co were 88.80%, 90.27%, 102.30%, and 105.55%, respectively.

### Statistical analyses

We used the mean ± standard deviation (SD), median (*P*_25_-*P*_75_), or percentiles to describe demographic characteristics and clinical indicators, and the chi-square test was used to compare categorical variables. Student’s *t* test was used to compare continuous variables that conformed to a normal distribution, and the Mann−Whitney *U* test was used to compare continuous variables that were not normally distributed between the case and control groups.

Generalized estimating equation (GEE) models were used to account for multiple IVF cycles in the same women according to previous studies (14, 15) to evaluate the associations of blood Cu, Zn, Se, and Co levels with the risk of EEA, and a binary distribution with a logit link function was used. In the above GEE models, Cu, Zn, Se, and Co levels were considered as continuous variables. Some covariates retained in the GEE models were selected based on the following criteria: reportedly related with the outcome or levels of Cu, Zn, Se, and Co or covariates that induced a >10% change in the main effect estimate in the unadjusted model. Finally, the following covariates were included in the adjusted models: cycle, age, BMI, education, duration of infertility, infertility diagnosis, passive smoking, and number of cycles. Active smoking and alcohol consumption before pregnancy were not included in the models because few infertile women reported smoking and drinking.

Because Cu, Zn, Se, and Co were highly correlated with each other and the relationship between these levels and EEA might not be linear, Bayesian kernel machine regression (BKMR) was used to flexibly model the joint effect of the mixture of Cu, Zn, Se, and Co levels on EEA and isolate single metal risk differences. BKMR could not only allow us to estimate nonlinear and nonadditive dose−response functions for a group of correlated metal exposures but also identify the contribution of every individual factor to the mixture effect; additionally, the form of the exposure–response function does not require *a priori* specification (16). In our present study, we modeled the exposure–response function as a weighted sum of Gaussian kernels. The following equation was employed: Y_i_ = h{Cu, Zn, Se, Co} + β^q^ Z_i_ + e_i_. Specifically, h{} was the exposure–response function of different exposure levels (Cu, Zn, Se, Co). The coefficient β^q^ was the effect estimates of the covariates. Z_i_ represented potential confounding factors that needed to be adjusted for, and e_i_ represented residuals. The model was run for up to 10,000 iterations. The combined effect of Cu, Zn, Se, and Co was estimated with all four metals at a specified threshold (25th, 30th, 35th,…, 75th percentile) compared to when the four metals were all set at the 50th percentile. The single effect of four metals was estimated by changing the levels of one metal from its 25th percentile to the 75th percentile while keeping the other three metals at their 25th, 50th, or 75th percentile. The univariate exposure–response function was employed when one metal was regarded as an independent continuous variable and EEA was regarded as a dependent binary outcome, while the other three metals were set at their medians. Finally, in the BKMR model, hierarchical variable selection was conducted to identify the metal with the greatest contribution within the mixture and displayed as the posterior inclusion probability (PIP).

All data were analyzed using SPSS for Windows (version 22.0; SPSS UK Ltd., Surrey, UK) or R (version 4.0.5, package ‘‘bkmr’’ and ‘‘ggplot2’’), and a two-sided *p* value <0.05 was considered statistically significant for all the tests unless otherwise indicated.

## Results

### Demographic and clinical characteristics

The demographic characteristics of 231 participants are shown in **Table 1**. The mean ± SD ages of women in the case and control groups were 33.28 ± 4.93 years and 32.04 ± 3.70 years, respectively, and the body mass index (BMI, calculated as body mass (kg) divided by height squared (m^2^), normal range: 18.5−24.9 kg/m^2^) was 22.43 ± 3.57 kg/m^2^ and 22.17 ± 3.12 kg/m^2^, respectively. Greater than 50% of women in both groups had higher education (college or above); 39.2% of cases and 42.7% of controls were exposed to secondhand smoke. The differences in the above characteristics were not significant between the two groups. In addition, other than diagnoses regarding causes of infertility, no significant differences were observed between the women of both groups with respect to women’s ages at menarche, their husbands’ ages, and sperm quality indices. With regard to levels of essential trace elements, the levels of Se of infertile women in the case group were lower than those in the control group (*p* = 0.034). No differences in Cu, Co, and Zn levels were noted between groups.

**Table 1:** Basic characteristics of cases and controls [Mean ± SD, Median (*P*_25_, *P*_75_) or n (%)].

Characteristics	Cases	Controls	*p-*Value
Maternal age (y)	33.28 ± 4.93	32.04 ± 3.70	0.056
Maternal BMI^a^ (kg/m^2^)			0.424
Underweight	7 (9.6)	11 (7.1)	
Normal	46 (63.0)	112 (72.3)	
Overweight	17 (23.3)	24 (15.5)	
Obesity	3 (4.1)	8 (5.2)	
Education			0.991
High school or below	40 (54.1)	84 (53.5)	
College	31 (41.9)	67 (42.7)	
University or above	3 (4.1)	6 (3.8)	
Duration of infertility (y)			0.846
< 5	58 (78.4)	128 (81.5)	
5 - 10	14 (18.9)	25 (15.9)	
> 10	2 (2.7)	4 (2.5)	
Passive smoking			0.669
No	45 (60.8)	90 (57.3)	
Yes	29 (39.2)	67 (42.7)	
Age at menarche (y)			0.561
≤12	8 (10.8)	12 (7.6)	
13–14	51 (68.9)	106 (67.5)	
15–16	14 (18.9)	32 (20.4)	
≥17	1 (1.4)	7 (4.5)	
Infertility diagnosis			<0.001
Pelvic adhesion, oviductal obstruction, etc.^b^	51 (68.9)	152 (96.8)	
DOR	11 (14.9)	2 (1.3)	
PCOS & EMT	10 (13.5)	2 (1.3)	
RSA	2 (2.7)	1 (0.6)	
Paternal age	34.18 ± 5.83	32.90 ± 4.87	0.083
Paternal sperm concentration (× 10^6^)^c^	40.55 (23.60, 82.83)	54.60( 32.15, 99.75)	0.053
Paternal sperm motility (%)^d^	36.10 (22.40, 54.40)	42.10 (29.58, 55.85)	0.075
Paternal sperm viability (%)^e^	59.65 (43.93, 78.10)	68.60 (52.68, 79.75)	0.181
Essential trace element concentration			
Cu (μg/L)	587.79 (540.75, 663.49)	572.73 (529.44, 653.35)	0.468
Zn (μg/L)	7231.98 (5981.56, 7854.43)	6715.91 (5601.17, 7645.23)	0.095
Se (μg/L)	93.54 (75.78, 105.07)	98.69 (83.80, 112.21)	0.034
Co (μg/L )	0.28 (0.19, 0.46)	0.25 (0.19, 0.36)	0.152

BMI, body mass index; DOR, diminished ovarian reserve; PCOS & EMT, polycystic ovarian syndrome & endometriosis; RSA, recurrent abortion;

a In the case group, one participant whose information about BMI was missing; in the control group, two participants whose information about BMI were missing;

b Pelvic adhesion, oviductal obstruction, etc. included the sequelae of pelvic inflammatory disease, scar uterus, chronic salpingitis, salpingectomy after ectopic pregnancy, unilateral tubal obstruction, and bilateral tubal obstruction;

c In the case group, six participants whose information about paternal sperm concentration were missing; in the control group, eight participants whose information about paternal sperm concentration were missing;

d In the case group, 26 participants whose information about paternal sperm viability were missing; in the control group, 35 participants whose information about paternal sperm viability were missing;

e In the case group, 11 participants whose information about paternal sperm motility were missing; in the controls group, 23 participants whose information about paternal sperm motility were missing.

The mean number of IVF cycles per woman was 1.7 in the case group (74 women with 123 cycles) and 1.1 in the control group (157 women with 180 cycles), as presented in **Table S1**. Therefore, the number of total cycles was 303 for all infertile women in the study. The clinical characteristics and cycle outcomes of the 231 participants (303 cycles) are shown in **Table S2**. All the clinical characteristics and cycle outcomes, including treatment protocol, COH outcomes, oocyte insemination technique, and *in vitro* fertilization outcomes, in the case group were significantly different from those in the control group.

We treated natural logarithm transformed levels of Cu, Zn, Se, and Co as continuous variables and fitted GEE models to assess the single effect of every element on the risk of EEA. A significantly positive association between Co concentration and EEA risk was found in both the unadjusted and adjusted models (OR = 1.72; 95% CI: 1.18−2.52 and OR = 2.27; 95% CI: 1.37−3.77, respectively). A significantly negative association between Se concentration and the risk of EEA was found in the unadjusted model (OR = 0.18; 95% CI 0.07−0.51); however, the significant association disappeared after adjusting for some confounders (OR = 0.32; 95% CI: 0.07−1.34). No significant association was noted between Cu or Zn and the risk of EEA. The results are presented in **Table 2**.

**Table 2:** OR [95% (CI)] for the associations of early embryonic arrest with concentrations of blood essential trace elements (Cu, Zn, Se, Co).

Metals	Model 1^a^		Model 2^b^	
	OR (95% CI)	*p*-value	OR (95% CI)	*p*-value
lnCu	1.18 (0.42, 3.33)	0.631	1.67 (0.46, 6.07)	0.433
lnZn	1.43 (0.50, 4.08)	0.503	3.36 (0.59, 19.14)	0.173
lnSe	0.18 (0.07, 0.51)	0.001	0.32 (0.07, 1.34)	0.118
lnCo	1.72 (1.18, 2.52)	0.005	2.27 (1.37, 3.77)	0.001

OR, odds ratio; CI, confidence interval; Cu, copper; Zn, zinc; Se, Selenium; Co, cobalt; ln, natural logarithm transformed.

a covariates included number of cycles.

b covariates included age, BMI, education, duration of infertility, infertility diagnosis, passive smoking and number of cycles.

**Figure 1:**
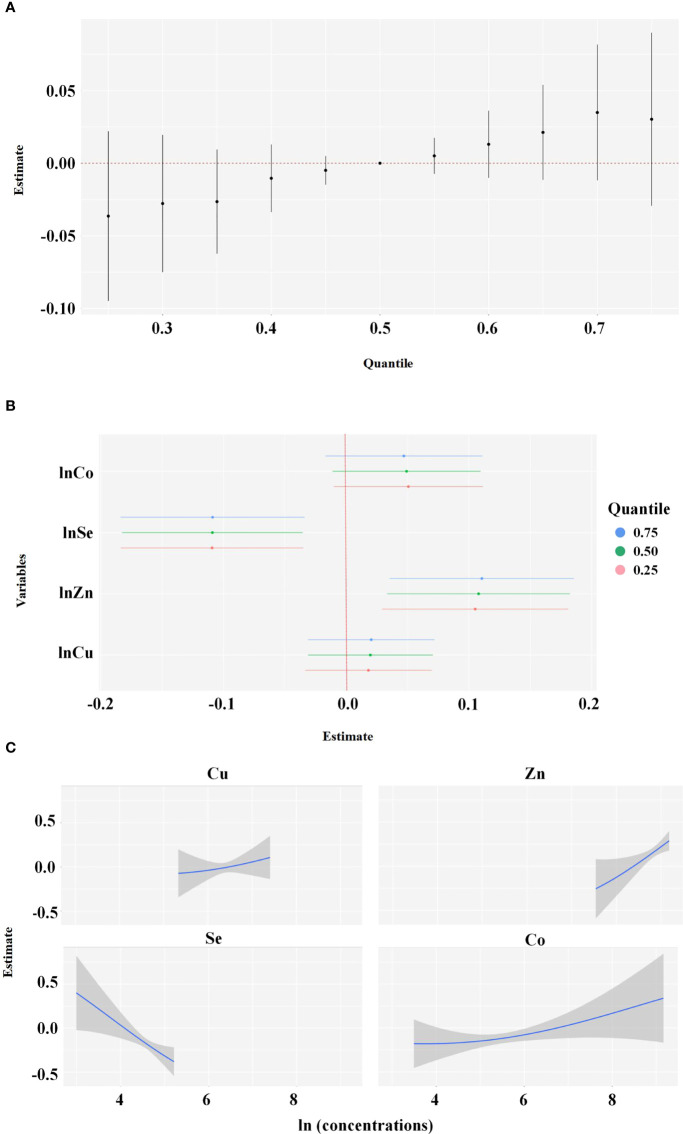
Joint effect of the Cu, Zn, Se, Co in blood on early embryonic arrest by Bayesian Kernel Machine Regression (BKMR) Model was adjusted for age, BMI, education, duration of infertility, infertility diagnosis, passive smoking and number of cycles. **(A)** overall effect of the Cu, Zn, Se and Co (estimates and 95% credible intervals). This plot compared the early embryonic arrest risk when Cu, Zn, Se and Co were at a particular quantile to when they were at the 50th percentile, respectively. **(B)** independent association of Cu, Zn, Se and Co (estimates and 95% credible intervals). This plot compared the early embryonic arrest risk when Cu, Zn, Se and Co was at the 75th of the metal concentrations with its 25th percentile, when concentrations of all the other metals were held at either the 25th (red line), 50th (green line), or 75th percentile (blue line). **(C)** Univariate exposure-response function and 95% confidence interval (grey part) for Cu, Zn, Se and Co when concentrations of other metals were hold at their median concentrations.

Spearman correlation analyses showed that the natural logarithm transformed levels of Cu, Zn, Se, and Co were highly correlated with each other (**Table 3**). Considering that these elements exist simultaneously in the human body, the BKMR model was used to assess the joint effect of the four-element mixture on the risk of EEA. **Figure 1** shows the results of the covariate-adjusted BKMR analyses for the risk of EEA. **Figure 1A** represents the overall effects of the four-element mixture, showing the estimated differences in odds ratios of EEA and 95% CIs when all element concentrations were held at a certain percentile compared to when all elements were held at their median concentrations. We did not observe an overall effect of the four-element mixture on the risk of EEA. Single-element risk differences of changing the concentration of a single element from the observed 25th percentile to the 75th percentile while holding other metals at their 25th, 50th, or 75th percentiles were also estimated. We found that only the 95% CI for the ORs of Se and Zn did not cross 0, and the risk of EEA decreased with increasing Se concentrations, whereas the risk of EEA increased with increasing Zn (**Figure 1B**). Based upon the estimated PIPs, BKMR identified Se (0.9506) as the most important contributor to the overall association followed by Zn, Co, and Cu (**Table 4**). **Figure 1C** shows the univariate exposure−response relationship and 95% CIs for one element and the risk of EEA based upon the estimated kernel function, controlling for the other three elements by holding them at their median concentrations. We found that the blood level of Se was negatively associated with the risk of early embryonic arrest, whereas Cu, Zn, and Co levels were positively related to the risk of early embryonic arrest.

**Table 3:** Correlations between ln-transformed concentrations of blood essential trace elements (Cu, Zn, Se, Co).

Metals	lnCu	lnZn	lnSe	lnCo
lnCu	1	-0.28**	0.07	0.26**
lnZn		1	0.55**	-0.19*
lnSe			1	-0.17*
lnCo				1

*indicates p value < 0.05; ** indicates p value < 0.001 **Table 4**.

**Table 4:** Posterior inclusion probabilities (PIP) of Ba, As, Pb, Hg by BKMR.

Metal	PIP
Cu	0.6712
Zn	0.8788
Se	0.9506
Co	0.7708

## Discussion

The results of this exploratory study indicated that the levels of essential trace elements (Cu, Zn, Se, and Co) might correlate with the risk of EEA to some extent. Although Co is an important constituent of vitamin B12 (17), higher Co levels in the human body can still lead to damage to various organs and tissues, such as the liver, kidney, pancreas, and heart, possibly through the generation of reactive oxygen species (ROS) and/or disturbance of DNA repair processes (17–21). To date, epidemiological studies on the association between Co levels in the human body and reproductive outcomes are very limited. In our study, the blood Co levels of women in the case group were not significantly higher than those in the control group. Additionally, we found that blood Co levels were positively associated with the risk of EEA only in the single-metal model, and the positive association no longer existed when all the other three metals (Cu, Zn, and Se) were fixed at the 25th, 50th, or 75th percentiles. Because blood Se level was negatively associated with the risk of EEA in infertile Chinese women in both the single-metal and BKMR models, the results implied that the harmful effect of Co on the risk of early embryonic development might be antagonized by the protective effect of Se.

A double-blind randomized intervention study including 120 female patients undergoing assisted reproductive techniques indicated that oral Se supplementation was significant in terms of obtaining good-quality embryos (22). Despite no statistically significant difference between the Se level and the risk of EEA in the adjusted single-metal model, our findings still showed that Se blood levels were negatively associated with the risk of EEA in Chinese infertile women, and the result was robust in the BKMR model. These findings implied that Se was beneficial for embryonic development. Additionally, women were always exposed to trace element mixtures rather than only one type of element. Element mixtures may have reciprocal effects on the risk of EEA, and Se was the most important element in the BKMR model. The results of the BKMR model were clearly more convincing. The beneficial effect of Se on reproduction might be because Se is a cofactor of antioxidative enzymes. These enzymes are responsible for the neutralization, elimination, and prevention of the synthesis of reactive oxygen species (ROS) (23, 24), and ROS could adversely affect the quality of female oocytes (25).

At present, there are few studies about the relationship between blood copper levels and human early embryonic development. Using a model of rat embryos *in vitro*, Jankovsky et al. demonstrated that copper is essential for early embryonic development (26). The results from a meta-analysis by Levandovska et al. showed that a decrease in serum copper levels at the beginning of pregnancy was associated with an increased risk of gestational hypertension (27). Prohaska and Brokate found that a specific amount of copper in the body had a protective effect during pregnancy (28). Excessive copper is harmful but rare given that it can lead to the formation of free radicals and destroy cell membranes and proteins in the body (29). Our study revealed no significant differences in blood copper levels between the two groups, which might explain why blood Cu levels were not associated with the risk of early embryonic arrest.

Zn is a cofactor of more than 3000 enzymes that regulate various cellular processes and cellular signaling; it is responsible for the DNA-binding ability of many transcription factors *via* the unique ability to form molecules known as Zn finger (Znf) proteins. Moreover, Zn is essential for cell division, differentiation, and the development of organs, such as the heart and kidney (30). Surprisingly, the results from our present study showed that when Cu, Se, and Co were fixed at the 25th, 50th, or 75th percentiles using BKMR models, Zn blood levels were positively correlated with the risk of EEA. Although the joint effect of these four elements on EEA was not significant, it still implies that infertile women should maintain appropriate levels of Cu, Zn, Se, and Co to avoid EEA.

To the best of our knowledge, this is the first study to explore the correlation between the risk of early embryonic arrest and Cu, Zn, Se, and Co blood levels in infertile women undergoing IVF in China. In addition, we investigated the associations using the single-element models and the BKMR model to select which element played a leading role. The results from both models showed that Se blood levels were negatively associated with the risk of early embryonic arrest in infertile Chinese women. This finding has important significance for the clinical prevention of early embryonic arrest among infertile women undergoing IVF. Finally, the majority of infertile women in the present study underwent at least one oocyte retrieval cycle; thus, selection bias was effectively avoided.

However, our study still has several limitations that should be interpreted carefully. One potential limitation is that the number of enrolled participants was relatively small, and the findings may be more convincing if a larger sample size was conducted. Another potential limitation is that the biological samples we used to measure the levels of Cu, Zn, Se, and Co were peripheral blood. In contrast, clinical laboratory protocols generally recommend analyzing serum Cu, Zn, Se, and Co levels to assess essential trace element status, including deficiency and overload conditions (31). However, some previous studies also used blood as a biological sample to assess the status of some essential trace elements in the human body (32, 33). Moreover, as a microenvironment composed of a serum ultrafiltrate, follicular fluid is in direct contact with a developing oocyte and its surrounding somatic cells (34, 35). Compared with blood, follicular fluid better reflects the internal environmental state (36). Another limitation of our study is that we measured the levels of these four elements at a single time point: on the day of oocyte retrieval when women initially began clinical treatment. However, our ability to assess the average cumulative status of these trace elements was limited in those patients who have undergone two or more oocyte retrieval cycles. Finally, this was a case−control study that could not infer a causal relationship between essential trace element status and the risk of EEA.

## Conclusion

We found that the levels of essential trace elements (Cu, Zn, Se, and Co) might correlate with the risk of EEA to some extent. The present study might provide a real-world perspective of the relationship between essential trace elements and the risk of EEA when considering them as a single element or as mixtures.

## Data availability statement

The original contributions presented in the study are included in the article/**Supplementary Material**. Further inquiries can be directed to the corresponding authors.

## Ethics statement

The studies involving human participants were reviewed and approved by The Biomedical Ethics Committee of Anhui Medical University. The patients/participants provided their written informed consent to participate in this study.

## Author contributions

YC, CL, and LS performed the study, analyzed the data, and drafted the manuscript. ZZ, TJ, DYL, and WZ contributed to the study conception. KZ and DL supervised the study. DJ, and YXC critically revised the manuscript and approved the final draft. All authors read and approved the final version of the manuscript.

## Funding

This work was supported by the National Natural Science Foundation of China (NSFC-U20A20350, NSFC-82173532, NSFC-81971455, NSFC-81871216, and NSFC-81601345), Postdoctoral Research Foundation of China (2021M700181), and Excellent Young Talents Fund Program of Higher Education Institutions of Anhui Province (gxyq2021173).

## Acknowledgments

The authors are grateful to the Scientific Research Center in Preventive Medicine, School of Public Health, Anhui Medical University for the technical support in our experiment. We are also very grateful to Yajing Liu for helping us.

## Conflict of interest

The authors declare that the research was conducted in the absence of any commercial or financial relationships that could be construed as a potential conflict of interest.

## Publisher’s note

All claims expressed in this article are solely those of the authors and do not necessarily represent those of their affiliated organizations, or those of the publisher, the editors and the reviewers. Any product that may be evaluated in this article, or claim that may be made by its manufacturer, is not guaranteed or endorsed by the publisher.
