# Supplementary material for: The association between essential trace element (copper, zinc, selenium, and cobalt) status and the risk of early embryonic arrest among women undergoing assisted reproductive techniques
Source: Front Endocrinol (Lausanne). 2022 Oct 26;13:906849. doi: 10.3389/fendo.2022.906849 (PMC9643704; doi:10.3389/fendo.2022.906849)
Supplement: Supplementary file 1 [file Table_1.doc]

**Supplementary Table 1**. Cycle information of women in the case and control group [n (%)].

| Cycle | n (%) | |
| --- | --- | --- |
|  | Controls | Cases |
| 1 | 157 (87.2) | 75 (61.0) |
| 2 | 19 (10.6) | 39 (31.7) |
| 3 | 4 (2.2) | 8 (6.5) |
| 4 | 0 (0) | 1 (0.8) |
| Total | 180 (100) | 123 (100) |
| Average of cycle | 1.1 | 1.7 |

**Supplementary Table 2.** Treatment protocols, cycle-specific characteristics, and in vitro culture outcomes from 303 in vitro fertilization cycles among 231 infertile women [Median (*P*_25_, *P*_75_) or n (%)].

| Characteristics | Cases | Controls | *p*-value |
| --- | --- | --- | --- |
| Treatment protocol |  |  | < 0.001 |
| Long GnRH agonist | 32 (27.4) | 107 (59.4) |  |
| Super long GnRH agonist | 5 (4.3) | 4 (2.2) |  |
| Short GnRH agonist | 6 (5.1) | 1 (0.6) |  |
| GnRH antagonist | 34 (29.1) | 62 (34.4) |  |
| Mild stimulation | 27 (23.1) | 4 (2.2) |  |
| Natural cycle | 12 (10.3) | 0 (0.0) |  |
| Others | 1 (0.9) | 2 (1.1) |  |
| COH outcomes |  |  |  |
| Dosage of Gn | 2050 (1350, 2700) | 2300 (1800, 2775) | 0.028 |
| Duration of Gn | 9 (8, 11) | 11 (10, 12) | < 0.001 |
| Day 3 FSH (IU/L) | 8.62 (6.63, 11.43) | 7.58 (6.50, 8.72) | 0.002 |
| Peak estradiol (pg/ml) | 3815.00 (2468.00, 7861.00) | 10088.00 (6609.00, 15467.00) | < 0.001 |
| Endometrial thickness | 10.40 (7.65, 12.00) | 11.30 (9.80, 13.10) | < 0.001 |
| AFC | 5.00 (3.00, 7.00) | 12.00 (8.00, 15.00) | < 0.001 |
| Oocyte insemination technique |  |  | < 0.001 |
| IVF | 68 (55.3) | 150 (83.3) |  |
| ICSI | 55 (44.7) | 30 (16.7) |  |
| In vitro fertilization outcomes |  |  |  |
| Oocytes retrieved | 3.00 (1.00,6.00) | 11.00(7.00,15.00) | < 0.001 |
| Mature (MII) oocytes | 2.00 (1.00, 4.00) | 9.00 (6.00, 13.00) | < 0.001 |
| Fertilization oocytes | 1.00 (1.00, 3.00) | 9.00 (6.00, 12.00) | < 0.001 |
| Cleavage embryo | 1.00 (1.00, 3.00) | 9.00 (6.00, 12.00) | < 0.001 |
| Normal (2PN) fertilized oocytes | 1.00 (0.00, 2.00) | 6.00 (4.00, 10.00) | < 0.001 |
| Normal (2PN) fertilized rate^a^ | 0.25 (0.00, 0.57) | 0.67 (0.50, 0.80) | < 0.001 |
| Day 3 blastomere number ≥ 6 | 0.00 (0.00, 1.00) | 6.00 (4.00, 10.00) | < 0.001 |
| Blastocyst | 0.00 (0.00, 0.00) | 4.00 (2.00, 7.00) | < 0.001 |

Note: GnRH, gonadotropin-releasing hormone; Gn, gonadotropin; COH, controlled ovarian hyperstimulation; FSH, follicle stimulating hormone; IU, international unit; AFC, antral follicle count; ICSI, intracytoplasmic sperm injection; IVF, in vitro fertilization; PN, pronucleus;

a, Normal (2PN) fertilisation rate is the number of oocytes with 2PN as a proportion of the number of oocytes retrieved.
